# Doxorubicin/Adriamycin Monotherapy or Plus Ifosfamide in First-Line Treatment for Advanced Soft Tissue Sarcoma: A Pooled Analysis of Randomized Trials

**DOI:** 10.3389/fonc.2021.762288

**Published:** 2021-11-22

**Authors:** Bi-Cheng Wang, Bo-Hua Kuang, Bo-Ya Xiao, Guo-He Lin

**Affiliations:** ^1^ Cancer Center, Union Hospital, Tongji Medical College, Huazhong University of Science and Technology, Wuhan, China; ^2^ Eastern Hepatobiliary Surgery Hospital, Second Military Medical University, Shanghai, China; ^3^ Department of Medical Psychology, Faculty of Psychology, Naval Medical University (Second Military Medical University), Shanghai, China; ^4^ Department of Oncology, The Second Affiliated Hospital of Anhui Medical University, Hefei, China

**Keywords:** soft tissue sarcoma, doxorubicin, ifosfamide, survival, tolerability

## Abstract

**Background:**

Doxorubicin/Adriamycin (ADM) alone or combined with ifosfamide (IFO) (AI) is available for previously untreated advanced soft tissue sarcoma (ASTS). However, the clinical choice between them remains controversial. In this pooled analysis, we comprehensively compared the efficacy and tolerability of AI versus ADM in patients with ASTS.

**Methods:**

PubMed, Web of Science, EMBASE, and Cochrane Library were systematically searched from inception to April 14, 2021. Eligible studies were randomized clinical trials comparing AI to ADM. The primary outcomes were overall survival (OS), progression-free survival (PFS), and objective response rate (ORR). Discontinuation rate (DR) and toxic death (TD) were explored as secondary outcomes.

**Results:**

Overall, three open-label randomized phase 2/3 clinical trials with a total of 1108 newly diagnosed ASTS patients were enrolled. Between AI and ADM, pooled hazard ratios were 0.93 (95% confidence interval 0.58-1.50, *p* = 0.78) for OS and 0.85 (0.57-1.25, *p* = 0.41) for PFS. While pooled risk ratios for ORR, DR, and TD were 1.37 (0.94-1.99, *p* = 0.10), 1.04 (0.74-1.46, *p* = 0.82), and 0.68 (0.19-2.36, *p* = 0.54) respectively. No publication bias was observed across the studies.

**Conclusion:**

In the first-line setting, adding IFO to ADM failed to benefit ASTS patients against ADM alone, even with comparable tolerability.

## Introduction

Anthracycline-based cytotoxic chemotherapies have been the main treatment of soft tissue sarcoma (STS) for nearly 40 years (1). Currently, the standard first-line treatments for advanced soft tissue sarcoma (ASTS) comprise doxorubicin/adriamycin (ADM) alone or combined with ifosfamide (IFO) (AI) (2). Both therapeutic strategies have been administrated in patients with rhabdomyosarcoma, clear cell sarcoma, angiosarcoma, dedifferentiated liposarcoma, undifferentiated pleomorphic sarcoma, synovial sarcoma, fibrosarcoma, leiomyosarcoma, and et al. However, if ADM monotherapy is adequate, might adding IFO to ADM be a necessity for ASTS?

In retrospective studies, AI combination therapy was found to be associated with better prognostics (3–6). A recently published report showed that the median progression-free survival (PFS) was, respectively, 8.2 months and 4.8 months with objective response rates (ORRs) of 19.5% and 25.6% for AI and ADM (7). Additionally, single-arm data have also indicated the feasibility and tolerability of AI (8–15).

Nevertheless, the effects brought by the addition of IFO to ADM remain controversial in the prospective studies (16–18). In this study, we conducted a pooled analysis to comprehensively compare the efficacy and tolerability between AI and ADM in patients with previously untreated ASTS.

## Materials and Methods

This analysis was conducted according to the Preferred Reporting Items for Systematic Reviews and Meta-analyses guideline (PRISMA) (19).

### Search Strategy

A literature search was systematically performed in PubMed, Web of Science, EMBASE, and Cochrane Library databases. The last searching date was April 14, 2021. Search keywords were (1) sarcoma, (2) doxorubicin or adriamycin, (3) ifosfamide, (4) first-line, and (5) trial or study. For more eligible studies, references of relevant records were reviewed.

### Selection Criteria

All of the eligible clinical trials should meet the following inclusion criteria: (1) patients were newly diagnosed as ASTS, (2) patients were treated with AI or ADM alone, (3) the efficacy and safety between the AI group and ADM group were compared, (4) enrolled studies were prospective clinical trials and published in English.

Exclusion criteria were (1) single-arm studies, (2) retrospective studies, (3) meeting abstracts, and (4) patients with Ewing’s sarcoma, osteosarcoma, malignant mesothelioma, chondrosarcoma, neuroblastoma, gastrointestinal stromal sarcoma, paraganglioma, primitive neuroectodermal tumor, or dermatofibrosarcoma protuberans. Any disagreements were resolved by discussion.

### Data Extraction and Quality Assessment

The primary outcomes were OS, PFS, and ORR, and the secondary outcomes were discontinuation rate (DR) and toxic death (TD). Bi-Cheng Wang and Bo-Hua Kuang independently extracted detailed data from the full articles and supplementary materials, including first author, journal, publication year, therapeutic regimens, number of patients, dosage, OS, PFS, response rate, DR, TD, and toxicities. For time-to-event data that were not reported or available directly, Engauge Digitizer software and the method reported by Jayne F Tierney were applied to extract and synthesize the survival data (20). Egger’s test was used to evaluate latent publication bias. Any discrepancies were resolved by consensus.

### Statistical Analysis

OS and PFS data were assessed by hazard ratio (HR) with 95% confidence interval (CI). While data of ORR, DR, and TD were evaluated by risk ratio (RR) with 95% CIs respectively. R (version 4.1) software and the “meta” package was used in the pooled analysis.

Both fixed effect and random effects models were calculated. t^2^ and *I* ^2^ statistic percentages were performed to test the heterogeneity. when *I* ^2^ < 50% or p value of heterogeneity < 0.1, pooled data through a fixed effect model with Mantel-Haenszel method were adopted. Otherwise, pooled data in the random effects model line were chosen. Differences with *p* values for all outcomes under 0.05 were considered statistically significant.

## Results

### Eligible Studies and Basic Characteristics

**Figure 1:**
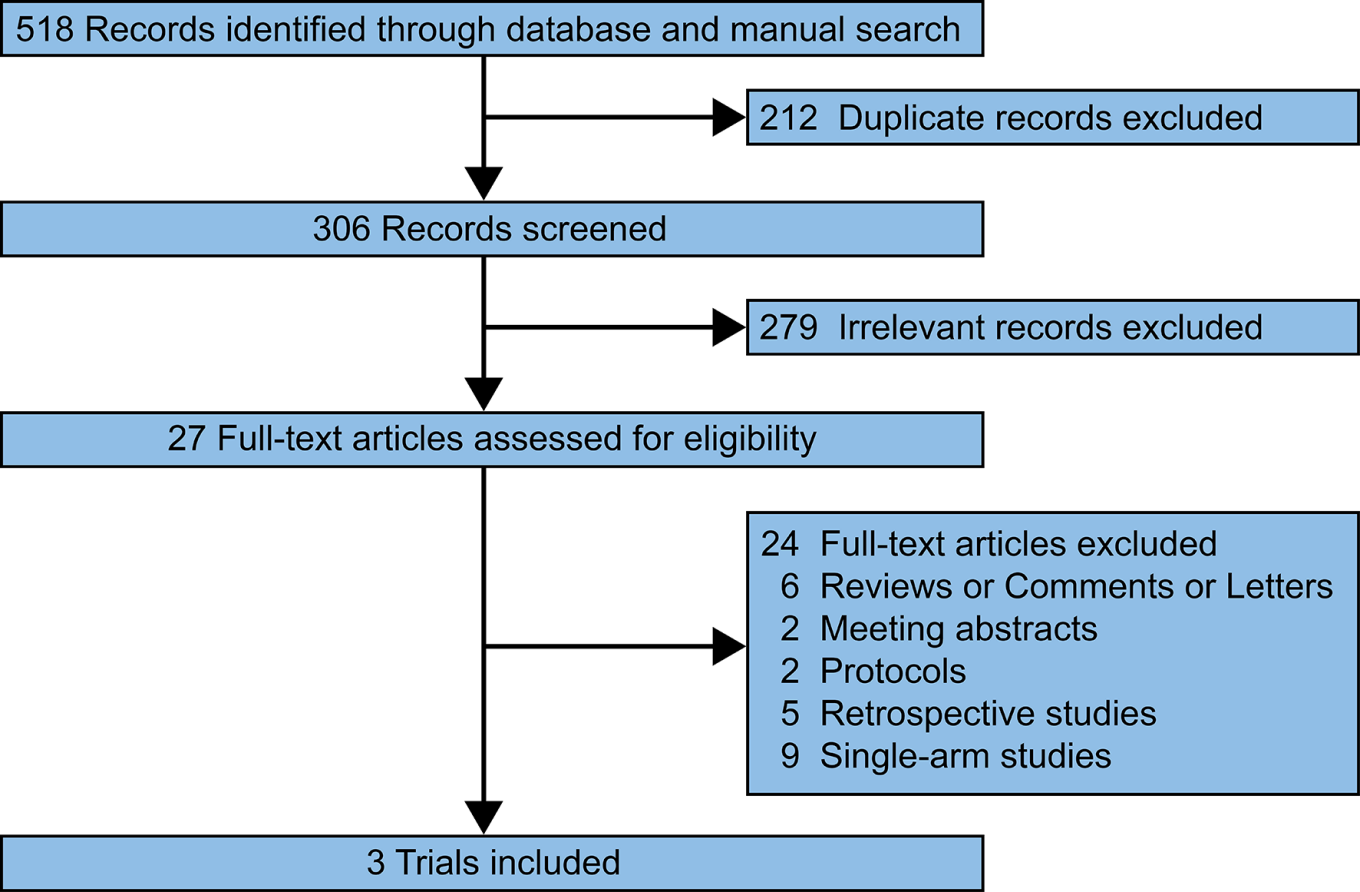
Flowchart of the selection process.

Our search of the PubMed, Web of Science, EMBASE, and Cochrane Library databases identified 518 relevant records. 211 duplicated records were removed. 279 records were excluded after screening the titles and abstracts. 24 full-text articles were excluded since the researches were reviews/comments/letters (n = 6), meeting abstracts (n = 2), protocols (n = 2), retrospective studies (n = 5), and single-arm studies (n = 9). Finally, three prospective trials were reviewed and pooled-analyzed (**Figure 1**) (16–18).

**Table 1** showed the basic characteristics of the enrolled studies. Two studies were open-label, randomized phase 3 trials (16, 18), and the other (17) was an open-label randomized phase 2 trial. Only the trial published by Ian Judson provided the trial number (NCT00061984).

**Table 1:** Basic characteristics of the eligible clinical trials.

First author	Phase	Journal	Publication year	Registered number	Groups	No. patents	Dose
Santoro et al. (16)	Open-label randomized phase 3 trial	Journal of Clinical Oncology	1995	NA	A	263	A: 75 mg/m^2^, every 3 weeks at least 2 cycles
					AI	258	A: 50 mg/m^2^ + I: 5 g/m^2^, every 3 weeks at least 2 cycles
Maurel et al. (17)	Open-label randomized phase 2 trial	Journal of Clinical Oncology	2009	NA	A	67	A: 75 mg/m^2^, every 3 weeks for 6 cycles
					AI	65	A: 90 mg/m^2^, every 2 weeks for 3 cycles + I: 12.5 g/m^2^, every 3 weeks for 3 cycles
Judson et al. (18)	Open-label randomized phase 3 trial	Lancet Oncology	2014	NCT00061984	A	228	A: 75 mg/m^2^, every 3 weeks for 6 cycles
					AI	227	A: 75 mg/m^2^ + I: 10 g/m^2^, every 3 weeks for 6 cycles

A, doxorubicin; I, ifosfamide; NA, not available.

In Joan Maurel’s trial, the ORRs were 23.4% in the ADM group and 24.1% in the AI group. Median PFS was 26 weeks and 24 weeks, respectively (17). In Ian Judson’s trial, no significant differences were found in OS between the groups (median OS: 12.8 months versus 14.3 months). But the median PFS and ORR were significantly higher for the combination therapy (7.4 months and 26%) versus the monotherapy (4.6 months and 14%) (18).

### Efficacy

HR and 95% CI data in Joan Maurel’s and Ian Judson’s trials could be extracted directly from the published articles. While the time-to-event data from Armando Santoro’s trial were reproduced according to the surviving curves. Comparing AI versus ADM, the reproduced HR for OS was 1.01 (95% CI 0.85-1.19), and for PFS was 0.94, (95% CI 0.81-1.10).

**Figure 2:**
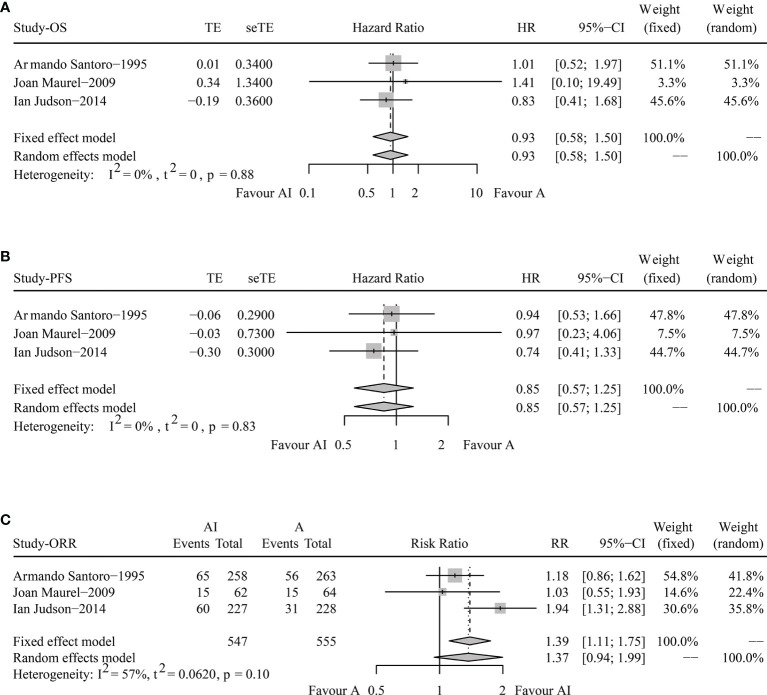
Forest plots of the pooled hazard ratio for overall survival **(A)** and progression-free survival **(B)** and odds ratio for objective response rate **(C)** between doxorubicin/adriamycin plus ifosfamide combination chemotherapy and doxorubicin/adriamycin monotherapy.

The pooled HR for OS were 0.93 (95% CI 0.58-1.50, Fixed effect model, *p* = 0.78). The forest plot indicated that patients obtained similar OS benefit from AI compared with ADM alone (**Figure 2A**).

The pooled HR for PFS were 0.85 (95% CI 0.57-1.25, Fixed effect model, *p* = 0.41), illustrating that AI combination therapy did not exhibit significant PFS superiority compared with ADM (**Figure 2B**).

The pooled analysis of ORR showed that the RR was 1.37 (95% CI 0.94-1.99, Random effects model, *p* = 0.1). No significant improvements in tumor responses were found when patients were treated with AI (**Figure 2C**).

### Tolerability

**Figure 3:**
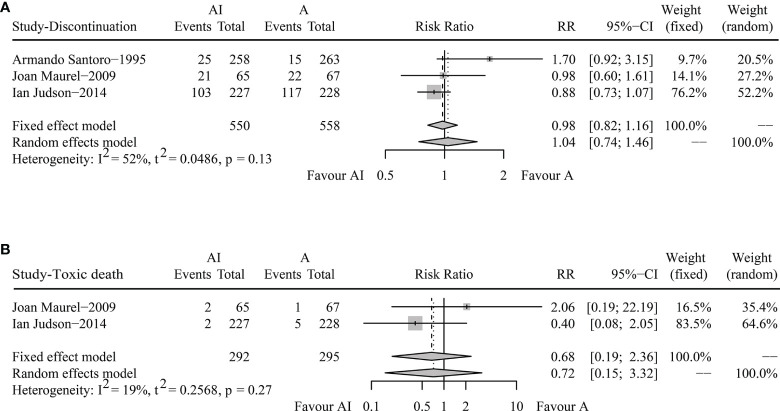
Forest plots of the pooled risk ratios for discontinuation rate **(A)** and toxic death **(B)** between doxorubicin/adriamycin plus ifosfamide combination chemotherapy and doxorubicin/adriamycin monotherapy.

In terms of DR, the overall DRs in AI and ADM groups were 27.1% and 27.6%. The pooled RR was 1.04 (95% CI 0.74-1.46, Random effects model, *p* = 0.82), demonstrating that both AI and ADM had comparable DRs (**Figure 3A**).

For toxic death, Joan Maurel reported two cases in the AI group and one in the ADM group, and Ian Judson recorded two in the combination chemotherapy group and five in the monotherapy group. The pooled RR was 0.68 (95% CI 0.19-2.36, Fixed effect model, *p* = 0.54) (**Figure 3B**). AI combination therapy did not increase the risk of death against ADM monotherapy.

### Risk of Bias

**Figure 4:**
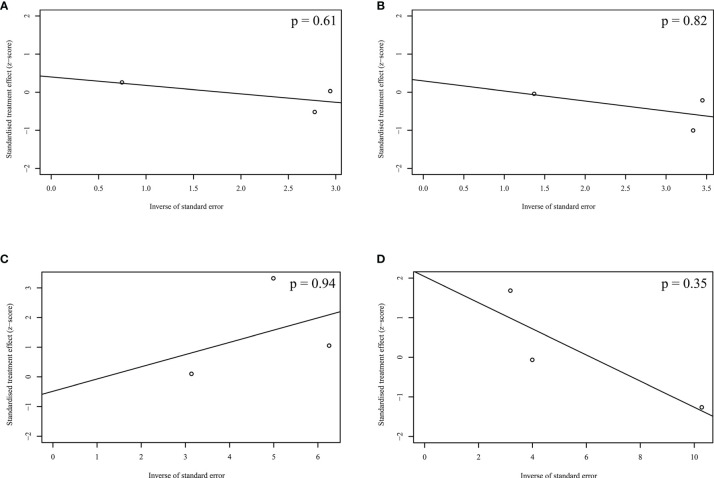
Publication bias acrossing the enrolled studies. **(A)** Overall survival; **(B)** Progression-free survival; **(C)** Objective response rate; **(D)** Discontinuation rate.

**Figure 4** showed the results of Egger’s test in the pooled analyses of OS, PFS, ORR, and DR. No publication bias among the studies was observed.

## Discussion

ADM is an anthracycline type of regimen, and IFO is an alkylating agent. Researchers combined the two drugs with different types to enhance the proapoptotic and antitumor activities (21, 22). However, according to our pooled analysis, AI combination chemotherapy had similar effects (OS, PFS, and ORR) and tolerability (DC and TD) compared with ADM alone. These results could provide a useful guide and constructive suggestions for clinicians.

In a previously published meta-analysis, the efficacy and toxicity of ADM monotherapy were evaluated compared to other first-line treatment choices (including vincristine, cyclophosphamide, actinomycin D, dacarbazine, ifosfamide, mitomycin C, cisplatin, trabectedin, melphalan, epirubicin, docetaxel, gemcitabine, pazopanib, and eribulin) in ASTS (23). The results showed that the efficacy between ADM alone and other first-line treatments was comparable, but higher risks of treatment-related toxicities were observed in the combination chemotherapy group. Therefore, the authors considered that ADM monotherapy might be preferred in the first-line setting of ASTS. But, in the study, ADM was compared to a total data of other first-line drugs, and this design might increase the bias. Thus, we should treat the conclusion with caution.

Due to the limited eligible studies, more detailed data, like pooled subgroup analysis, could not be further analyzed. Actually, there should be some specific populations who could benefit from the AI combination chemotherapy. In the post-hoc analysis of Joan Maurel’s trial, poor performance status or lung metastasis alone predicted worse OS and PFS (17). In the other study reported by Ian Judson, 40-49 years old or non-liver metastasis patients could benefit more from AI combination therapy (18). Accordingly, we suspect that patients under 50 years old with well performance and without lung metastasis might be suggested to receive AI combination therapy.

Although DR and TD were comparable between both groups, treatment-related hematological and non-hematological toxicities deserved our attention. AI showed a higher incidence of myelosuppression. In Armando Santoro’s study, 32% of patients in the AI group and 13% in the ADM group had experienced grade 4 leukopenia (16). Additionally, in Ian Judson’s study, AI chemotherapy caused more leucopenia (43% versus 18%), anemia (35% versus 4%), and thrombocytopenia (33% versus <1%) compared to ADM monotherapy (18). In terms of non-hematological adverse events, Joan Maurel reported that the incidences of asthenia and mucositis were more frequent when patients were treated with AI (17). On the other hand, TD had been recorded in both treatment groups (four of 292 in the AI and six of 295 in the ADM), indicating that great cautions should be paid during the whole treating process.

Cumulative cardiotoxicity is another serious side-effect of ADM. Among the eligible studies, only Armando Santoro reported more frequent of cardiotoxicity in the combination group. Since cardiotoxicity of ADM is dose-limiting, pegylated-liposomal ADM, a methoxypoly liposomes coated formulation of ADM, could effectively prolong half-life in blood and reduce cardiotoxicity (24). In Liu’s single-arm study (25), newly diagnosed ASTS patients had received pegylated liposomal-AI. The results showed that the median OS was 24 months, the ORR was 26.1%, and no grade three or more cardiotoxicity was reported. Moreover, a case study reported that pegylated-liposomal ADM could be an efficient second-line treatment of ASTS with recurrence after ADM therapy (26). These results demonstrated that pegylated-liposome ADM could be an active and well-tolerated therapeutic drug in treating naive ASTS patients.

### Limitations

Several limitations existed in this study. (1) The enrolled studies were open-label trials, which might bias disease progression and response assessment. (2) The doses of ADM and IFO differed from each study that might also increase the bias.

## Conclusion

Although both therapies had similar DC and TD, adding IFO to ADM failed to improve the efficacy (including OS, PFS, and ORR) compared to ADM alone in treatment-naive ASTS patients. Therefore, as a first-line strategy, ADM monotherapy could be adequate. More future prospective studies are warranted to confirm our results.

## Data Availability Statement

The original contributions presented in the study are included in the article/supplementary material. Further inquiries can be directed to the corresponding author.

## Author Contributions

Study design: B-CW. Data extraction: B-CW and B-HK. Data analysis: B-CW and B-HK. Manuscript writing and edition: B-CW, B-YX, and G-HL. All authors contributed to the article and approved the submitted version.

## Funding

This study was supported by the Hubei Provincial Natural Science Foundation (Grant number: 2020CFB397 to B-CW) and the Independent Innovation Foundation of Wuhan Union Hospital (Grant number: 2019-109 to B-CW).

## Conflict of Interest

The authors declare that the research was conducted in the absence of any commercial or financial relationships that could be construed as a potential conflict of interest.

## Publisher’s Note

All claims expressed in this article are solely those of the authors and do not necessarily represent those of their affiliated organizations, or those of the publisher, the editors and the reviewers. Any product that may be evaluated in this article, or claim that may be made by its manufacturer, is not guaranteed or endorsed by the publisher.
